# General practitioners perceptions on advance care planning for patients living with dementia

**DOI:** 10.1186/s12904-015-0019-x

**Published:** 2015-04-23

**Authors:** Kevin Brazil, Gillian Carter, Karen Galway, Max Watson, Jenny T van der Steen

**Affiliations:** School of Nursing and Midwifery, Queen’s University Belfast, 97 Lisburn Road, Belfast, BT9 7BL Ireland; Northern Ireland Hospice, 18 O’Neill Road, Newtownabbey, BT36 6WB Ireland; Department of General Practice & Elderly Care Medicine, VU University Medical Center Amsterdam, EMGO Institute for Health and Care Research, PO Box 7057, Amsterdam, 1007 MB The Netherlands

**Keywords:** Advance care planning, Communication, Decision-making, Dementia, General practice

## Abstract

**Background:**

Advance care planning (ACP) facilitates communication and understanding of preferences, nevertheless the use of ACPs in primary care is low. The uncertain course of dementia and the inability to communicate with the patient living with dementia are significant challenges for GPs to initiate discussions on goals of care.

**Methods:**

A cross-sectional survey, using a purposive, cluster sample of GPs across Northern Ireland with registered dementia patients was used. GPs at selected practices received the survey instrument and up to four mail contacts was implemented.

**Results:**

One hundred and thirty-three GPs (40.6%) participated in the survey, representing 60.9% of surveyed practices. While most respondents regarded dementia as a terminal disease (96.2%) only 37.6% felt that palliative care applied equally from the time of diagnosis to severe dementia. While most respondents thought that early discussions would facilitate decision-making during advanced dementia (61%), respondents were divided on whether ACP should be initiated at the time of diagnoses. While most respondents felt that GPs should take the initiative to introduce and encourage ACP, most survey participants acknowledged the need for improved knowledge to involve families in caring for patients with dementia at the end of life and that a standard format for ACP documentation was needed.

**Conclusion:**

Optimal timing of ACP discussions should be determined by the readiness of the patient and family carer to face end of life. ACP discussions can be enhanced by educational strategies directed towards the patient and family carer that enable shared decision-making with their GP when considering options in future care.

**Electronic supplementary material:**

The online version of this article (doi:10.1186/s12904-015-0019-x) contains supplementary material, which is available to authorized users.

## Background

Chronic illness accounts for most deaths in the industrialized world. Heart disease, stroke and dementia are the leading causes of death [1]. Between 1990 and 2040, annual neurodegenerative disease mortality is projected to increase between 119% and 231%. The major part of this increase is from deaths attributed to dementia [1].

The provision of palliative care for dementia presents unique challenges. Although variation exists, survival time for individuals diagnosed with dementia ranges from 3 to 10 years [2,3]. Two significant factors for families and General Practitioners (GPs) are the uncertain course of the disease, as well as difficulty communicating with the patient in the terminal and most symptomatic stages, when communication would be most helpful. As the disease progresses to the terminal stage, the ability for the person with dementia to meaningfully communicate, ambulate or manipulate objects is severely impaired, if not impossible [4].

Despite the poor prognosis of persons with dementia coupled with their growing numbers their health care preferences are not always known [5-7]. Often, this leads to difficult decision-making for family members. Study findings have indicated that health care providers may default to full treatment when an individual’s care preferences are unknown, and consequently people with dementia have received burdensome interventions [7-9].

Advance Care Planning (ACP) is a process that facilitates the communication and understanding of care preferences between a person deemed to have decision-making capacity and their primary health care provider, family members or surrogate decision maker [10,11]. Cantor and Pearlman [12] assert that ACP involves three components: the consideration of health care options and expression of the person’s values; communicating their wishes, and subsequent documentation. In England and Wales the Mental Capacity of Act (MCA) [13], designed to protect people who can’t make decisions for themselves or lack the mental capacity to do so, provides a statutory framework where ACP must be used and not relegated to a practice option. While the MCA does not extend to Scotland and Northern Ireland, regulatory frameworks do exist in those jurisdictions with the aim to protect the interests of those who do not have decisional capacity to manage their affairs and personal care. While legislation such as the MCA is important its incorporation into practice can be problematic implying uneven application across settings [14].

Good communication is at the heart of good dementia care. Good dementia care will engage the patient and their family in decision-making as far as possible. Nevertheless, the use of ACPs in the primary care setting is low [15]. This is further hindered by reports that physicians are reluctant to discuss ACPs with patients [15-17].

The purpose of this study was to describe the attitudes and practice preferences of GPs working within the UK’s National Health System (NHS) regarding communication and decision-making for patients with dementia and their families.

## Method

### Design and sample

The study conducted a cross-sectional postal survey of GPs located across Northern Ireland. A purposive, cluster sampling approach was used to target GPs with responsibility for patients with dementia. The Quality and Outcomes Framework (QOF) data (http://www.dhsspsni.gov.uk/dementia_indicators_by_practice_suppressed_2011-12.pdf) includes incidence and prevalence figures for dementia at the GP practice level. In an effort to select GP practices with experience of dementia care, the codes of those practices that indicated a prevalence of 30 or more patients diagnosed with dementia in the past year (2011–2012) were selected for inclusion to the study. The practice codes listed in the publicly available QOF data were matched to practice names using the Business Services Organization’s Practice and GP lists, also containing practice codes (http://www.hscbusiness.hscni.net/services/1816.htm). A simple matching process was used to create a practice-level sampling frame. GPs at the selected practices received a personalized self-complete postal survey. The sample comprised 340 GPs representing 174 practices (49% of all practices in Northern Ireland). Multiple contacts are essential for maximizing response to mail surveys [18], therefore a system of up to four mail contacts was implemented. As an incentive to complete the survey a prize draw was held where respondents would have the opportunity to win an iPad mini.

### Instrument

The “Care for Dementia Patients at the End of Life” survey instrument explores GPs’ perceptions on palliative care for individuals with dementia (Additional file 1). The items included in the instrument were based on recommendations proposed by the European Association for Palliative Care (EAPC) on palliative care in dementia [19], and was pretested on a sample of palliative care physicians and GPs. The survey included a quantitative evaluation of perceptions of dementia as a terminal illness; communication; ACP, and also decision-making. An evaluation of the importance, the perceived barriers, and challenges of addressing the barriers regarding twelve elements of palliative care in dementia based on the eleven domains of palliative care in dementia put forward by the EAPC was also included. Following this quantitative evaluation, we solicited respondents’ suggestions of the three most significant barriers to the provision of palliative care in dementia and associated potential solutions, which could be related or unrelated to the quantitative evaluation of elements of palliative care. Finally, the survey recorded respondent characteristics.

### Data management and analyses

In this paper we report on respondents’ perceptions on communication, ACP and decision-making. Questions asked participants to select one response within a 5-point scale (strongly disagree; moderately disagree; neither agree nor disagree; moderately agree, and strongly agree). A ‘don’t’ know’ response choice was added to the instrument. Participants that indicated they neither agree nor disagree with the statement were categorized as ‘reserving judgement’. Responses to each survey item were graphed and frequencies examined using means (standard deviations). Survey data were inputted and managed in IBM SPSS Statistics 21.

### Ethical approval

Research Ethics was obtained through the Research Ethics Committee at the School of Nursing and Midwifery, Queen’s University Belfast. Consent was implied by the receipt of a completed questionnaire.

## Results

A total of 138 responses were received, of these 133 provided completed surveys, of the remaining five responses, four respondents indicated that they were too busy to complete the survey and one respondent submitted their completed survey after data analyses, giving a response rate of 40.6% (138/340) , representing 60.9% (106/174) of the surveyed practices. Table 1 describes the characteristics of the respondents revealing that their mean age was 49.3 years, with over half being male (57.4%).

**Table 1:** **Demographics of physician survey respondents**

**Characteristic**	**n** ^*^	
Gender (% male)	129	57.4
Age (years) (mean [SD])	126	49.3 [8.3]
Years in practice (mean [SD])	128	24.7 [8.0]
Time spent providing clinical care (FTE) (median [range])	126	1.00 [0.6]
Practice time spent providing clinical care in nursing home (n [%])	129	
<10%		74 [57.4]
10%-24%		51 [39.5]
25%-49%		3 [2.3]
50%-74%		1 [0.8]
≥75%		0
Frequency of visits for a typical nursing home patient (n [%])	128	
At least daily		4 [3.1]
At least weekly		62 [48.4]
At least monthly		25 [19.5]
Every 2 months		22 [17.2]
Every 6 months		10 [7.8]
Less than every 6 months		5 [3.9]
Estimated number of dying dementia patients cared for in past year (n [%])	129	
None		1 [0.8]
1 to 4		60 [46.5]
5 to 9		43 [33.3]
10 to 19		18 [14.0]
20 or more		7 [5.4]

*The number of completed responses out of 133.

### Informing patients and families

Table 2 reports on GPs’ perceptions on the impact of informing patients and families around the time of diagnosis on what severe dementia looks like. Most respondents agreed (strongly or moderately) with the statement that early discussions would facilitate decision-making during the more advanced stages of dementia, as the family would be better prepared (Statement a). It is noteworthy that 31% held reservations on the value of holding these discussions at the time of diagnosis. A large number of respondents did acknowledge that initiating this discussion involved the risk of unnecessarily increasing anxiety amongst patients and families (Statement d). Most respondents did not associate increased anxiety among patients and families with requests for inappropriate use of pain medications (Statement b) or requests for hastening death (Statement c).

**Table 2:** **Physician agreement with statements describing the process of informing patients and families about what dementia looks like at the time of diagnosis (n [%])**

**Statement**		**n** ^*^	**Strongly disagree**	**Moderately disagree**	**Neither agree nor disagree**	**Moderately agree**	**Strongly agree**	**Don’t know**
a	Facilitates later decision-making because families are better prepared	132	3 [2.3]	11 [8.3]	27 [20.5]	60 [45.5]	31 [23.5]	0
b	Will increase requests for inappropriately high levels of pain relieving medication	131	48 [36.1]	53 [39.8]	22 [16.5]	7 [5.3]	1 [0.8]	2 [1.5]
c	Will increase requests for hastening death	131	49 [36.8]	46 [34.6]	29 [21.8]	7 [5.3]	0	2 [1.5]
d	Will increase patients’ and families’ anxiety unnecessarily	133	11 [8.3]	26 [19.5]	21 [15.8]	46 [34.6]	29 [21.8]	0
e	Is not needed because families will witness patient’s decline later and this will sufficiently facilitate decision-making	133	32 [24.1]	48 [36.1]	20 [15.0]	22 [16.5]	11 [8.3]	0
f	Is not necessary as most patients will not progress to severe dementia	132	37 [28.0]	55 [41.7]	24 [18.2]	15 [11.4]	1 [0.8]	0

*The number of responses refers to those giving some level of disagreement/agreement.

### Advance care planning

Table 3 reveals that respondents were divided on the statement that ACP should be initiated at the time of diagnoses (Statement a). However, most respondents did feel that initiating ACP should be determined by the patients’ and families’ willingness to face the end of life (Statement j). Advance Directives, increasingly known as Advance Decisions, describe the documented statement explaining what medical treatment the individual would want in the future, should the individual ‘lack capacity’. In our survey only 51.1% (n = 68) of respondents moderately or strongly agreed with the statement that an Advance Directive was essential when a patient cannot participate in treatment decisions (Statement c). While most respondents felt the physician should take the initiative to introduce and encourage ACP (Statement d), most respondents felt that family members should not simply agree with the physician on the goals of care (Statement e). GPs’ responses were widely distributed on their appraisal of the success of the ACP process when family members have difficulty in understanding the limitations and complications of life sustaining therapies, or could not accept their loved one’s prognoses (Statements f and g). Most respondents did report that GPs need training to improve their knowledge to successfully involve families in caring for dementia at the end of life (Statement i) and that there should be an agreed format for ACPs (Statement h) (Table 4).

**Table 3:** **Physician agreement with statements describing ACP about future care at the end of life (n [%])**

**Statement**		**n** ^*^	**Strongly disagree**	**Moderately disagree**	**Neither agree nor disagree**	**Moderately agree**	**Strongly agree**	**Don’t know**
a	Advance care planning on end of life care should be initiated at the time of diagnosis of dementia	133	20 [15.0]	41 [30.8]	19 [14.3]	41 [30.8]	12 [9.0]	0
b	The process of advance care planning should involve revisiting plans with the patient and the family on a highly frequent basis	133	11 [8.3]	47 [35.3]	11 [8.3]	44 [33.1]	20 [15.0]	0
c	When a patient cannot participate in treatment decisions an advance directive is essential	132	9 [6.8]	21 [15.8]	34 [25.6]	51 [38.3]	17 [12.8]	1 [0.8]
d	The physician should take the initiative to introduce and encourage advance care planning	133	1 [0.8]	4 [3.0]	18 [13.5]	65 [48.9]	45 [33.8]	0
e	The advance care planning process requires my making family members agree with the physician on goals of care	133	25 [18.8]	45 [33.8]	26 [19.5]	30 [22.6]	7 [5.3]	0
f	When family members have difficulty understanding the limitations and complications of life sustaining therapies, the physician cannot successfully guide the advance care planning process	132	4 [3.0]	47 [35.3]	26 [19.5]	46 [34.6]	9 [6.8]	1 [0.8]
g	When the physician cannot make family members accept their loved one’s prognosis, the advance care planning process fails	130	7 [5.3]	47 [35.3]	35 [26.3]	35 [26.3]	6 [4.5]	3 [2.3]
h	There should be an agreed format for advance care plans	132	1 [0.8]	2 [1.5]	9 [6.8]	67 [50.4]	53 [39.8]	1 [0.8]
i	Physicians need improved knowledge to successfully involve families in caring for dementia patients at the end of life	133	1 [0.8]	6 [4.5]	20 [15.0]	65 [48.9]	41 [30.8]	0
j	The pace of advance care planning is primarily determined by patient’s and family’s willingness to face the end of life	132	1 [0.8]	11 [8.3]	19 [14.3]	64 [48.1]	37 [27.8]	1 [0.8]
k	Families and patients who are involved in advance care planning should become informed about commonly occurring health problems associated with severe dementia, such as pneumonia and intake problems	133	0	2 [1.5]	2 [1.5]	62 [46.6]	67 [50.4]	0
l	In the case of increasing severity of dementia, the patient’s best interest may be increasingly served with a primary goal of maximizing comfort	133	1 [0.8]	0	1 [0.8]	24 [18.0]	107 [80.5]	0

*The number of responses refers to those giving some level of disagreement/agreement.

**Table 4:** **Physician agreement with statements describing decision-making (n [%])**

**Statement**		**n** ^*^	**Strongly disagree**	**Moderately disagree**	**Neither agree nor disagree**	**Moderately agree**	**Strongly agree**	**Don’t know**
a	Shared decision making including the patient and family caregiver as partners should be a clinical practice goal	132	0	0	4 [3.0]	46 [34.6]	82 [61.7]	1 [0.8]
b	The health care provider should always prioritize the patient’s needs in decision- making	133	0	4 [3.0]	7 [5.3]	39 [29.5]	83 [62.4]	0
c	The physician should be responsible for making the final decision on the patient’s needs	133	14 [10.5]	40 (30.1)	48 [36.1]	23 [17.3]	8 [6.0]	0

*The number of responses refers to those giving some level of disagreement/agreement.

### Decision-making

On the topic of shared decision-making most respondents agreed with the statement that shared decision-making including the patient and family caregiver as partners, should be a clinical practice goal (Statement a), and that the health care provider should always prioritize the patient’s needs in decision-making (Statement b). Interestingly, respondents were more divided on the statement that physicians should be responsible for making the final decision on the patient’s needs (Statement c) (Table 4).

## Discussion

The study reported here is unique, insofar as no survey has been undertaken within GP practices in the UK addressing the topic of ACP for individuals living with dementia. While most respondents in our study agreed that having discussions in the early stages of diagnoses would facilitate decision-making during the advanced stages of dementia, a sizable number of respondents held reservations that these discussions should be held at the time of diagnoses. Most respondents preferred that the optimal timing of ACP discussions be determined by the readiness of the patient and family to acknowledge end of life considerations and that these discussions should be initiated by the GP. This approach stresses the importance on the relationship the GP has with the patient and their family carers so that they can consider the timing to explore patients’ and families’ perceptions of prognosis and willingness to discuss these issues.

GPs in this survey supported the importance of additional training on how to engage family carers in caring for dementia patients at the end of life. While most respondents viewed shared decision-making with the patient and family carer as a clinical practice goal in ACP discussions, a barrier acknowledged by a number of respondents was the difficulty family members have in accepting the prognoses of the family member living with dementia, and the difficulty patients’ and families’ have in understanding the limitations of complications of life sustaining therapies. This family related barrier to having ACP discussions stresses the importance of educational strategies to assist patients and family carers in being informed about the disease trajectory of dementia and common health problems associated with it. Educational materials to address this issue should include information about understanding the trajectory of dementia at the end of life and the role of comfort care measures [20-22]. However educational material alone has not been found to be effective as when used in combination with other interventions such as discussion sessions over multiple visits with a trained facilitator [23]. Future research is necessary to assess methods of facilitating ACP discussions that address the barriers identified by respondents in this survey.

Limitations of the study are worth noting. The study sample of GPs was limited to Northern Ireland. While the findings provide insight on GP perceptions in this region, caution is advised to applying findings to the rest of the UK. As part of our sampling strategy we sought GP practices that had a familiarity with the issue we were examining and thus selected those practices that indicated a prevalence of 30 or more patients diagnosed in the past year. Consequently, GP practices less focused on dementia were not included; their inclusion in this study may have revealed more variation in response to the issues that we examined. Despite vigorous efforts to generate a robust response rate a low individual GP response rate is noted. Evidence of falling questionnaire response rates of GPs has been identified in research to be affiliated to full work schedules [24,25]. However, most practices included in the survey did participate offering strong representation at the practice level. Despite the best efforts of total survey design, non-response bias is still likely to persist.

## Conclusions

In our study most respondents indicated a willingness to discuss ACP with the view that the optimal timing of ACP discussions should be determined by the readiness of the patient and the family carer, and that these discussions should be initiated by the GP. The present study supports the importance of educational strategies directed towards the patient and family carer that enables shared decision-making with their GP when considering options in future care. Further, GPs acknowledged the need for continued training on how to effectively engage family carers in dementia care.

## Additional file

Additional file 1:
**Care for Patients with Dementia at the End of Life.**
