# Supplementary material for: General practitioners perceptions on advance care planning for patients living with dementia
Source: BMC Palliat Care. 2015 Apr 23;14:14. doi: 10.1186/s12904-015-0019-x (PMC4410576; doi:10.1186/s12904-015-0019-x)
Supplement: Additional file 1: — Care for Patients with Dementia at the End of Life. [file 12904_2015_19_MOESM1_ESM.doc]

**Care for Patients with Dementia at the End of Life**

A survey about physicians’ priorities in caring for people with dementia at the end of life

Principle Investigator

Professor Kevin Brazil

Professor of Palliative Care

School of Nursing and Midwifery

Queen’s University Belfast

Co-Investigators

Dr Jenny Van der Steen

Senior Researcher

VU University Medical Centre Amsterdam

Dr Karen Galway

Lecturer Mental Health

School of Nursing and Midwifery

Queen’s University Belfast

Professor Max Watson

Visiting Professor University of Ulster

Medical Director Northern Ireland Hospice

Honorary Consultant Princess Alice Hospice, Esher


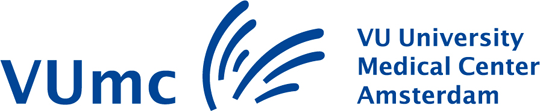


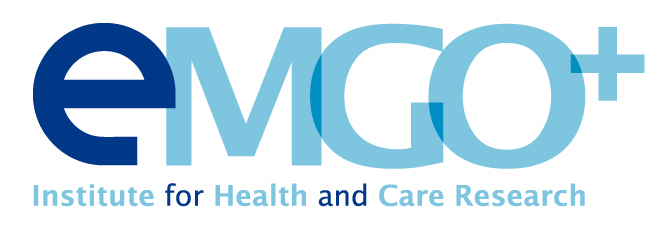


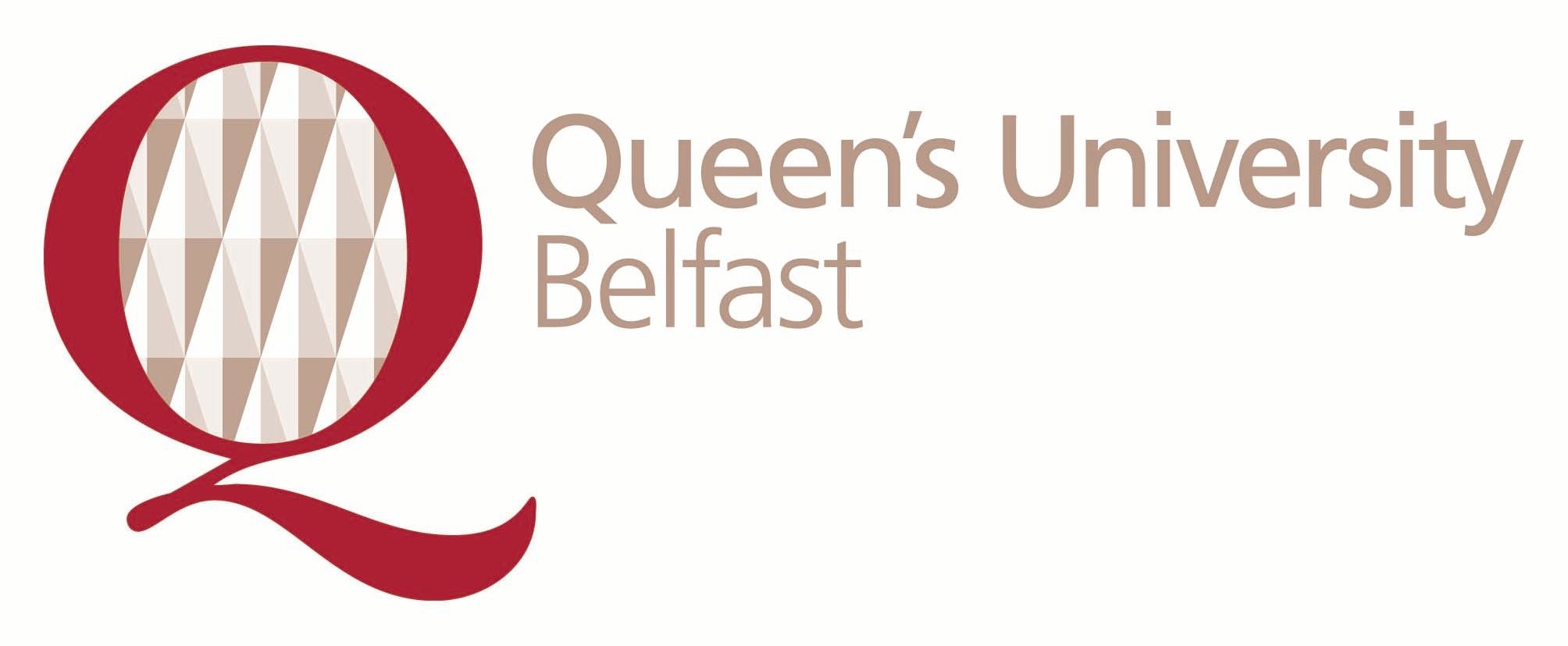


**Care for Patients with Dementia at the End of Life**

08

**Fall**

Increasingly, people are dying with or from dementia. This survey study is about physicians’ priorities in caring for people with dementia at the end of life. The items are based on a proposed set of European guidelines. Your opinions can help shape future policy and practice. We count on your contribution, thank you very much in advance for this.

**Section A: Statements**

Please rate your level of agreement with the following statements by circling the appropriate number in each row.

|  | **Strongly disagree** | **Moderately disagree** | **Neither agree nor disagree** | **Moderately agree** | | **Strongly agree** | **Don’t know** |
| --- | --- | --- | --- | --- | --- | --- | --- |
|  | | | | | | | |
| 1. **Dementia and the End of Life** | | | | | | | |
|  | | | | | | | |
|  | | | | | | | |
| 1. Dementia can be regarded as a disease you can die from | 1 | 2 | 3 | 4 | | 5 | 0 |
|  | | | | | | | |
| 1. Palliative care applies equally from the time of diagnosis to the stage of severe dementia | 1 | 2 | 3 | 4 | | 5 | 0 |
|  | | | | | | | |
|  | | | | | | | |
| 1. **Informing Patients and Families** | | | | | | | |
|  | | | | | | | |
|  | | | | | | | |
| **Informing patients and families around the time of diagnosis on what severe dementia looks like:** | | | | | | | |
|  | | | | | | | |
| 1. Will increase patients’ and families’ anxiety unnecessarily | 1 | 2 | 3 | | 4 | 5 | 0 |
|  | | | | | | | |
| 1. Facilitates later decision-making because families are better prepared | 1 | 2 | 3 | | 4 | 5 | 0 |
|  | | | | | | | |
| 1. Is not needed because families will witness patient’s decline later and this will sufficiently facilitate decision-making | 1 | 2 | 3 | | 4 | 5 | 0 |
|  | | | | | | | |
| 1. Is not necessary as most patients will not progress to severe dementia | 1 | 2 | 3 | | 4 | 5 | 0 |
|  | | | | | | | |
| 1. Will increase requests for inappropriately high levels of pain relieving medication | 1 | 2 | 3 | | 4 | 5 | 0 |
|  | | | | | | | |
| 1. Will increase requests for hastening death | 1 | 2 | 3 | | 4 | 5 | 0 |

|  | **Strongly disagree** | **Moderately disagree** | **Neither agree nor disagree** | **Moderately agree** | **Strongly agree** | **Don’t know** |
| --- | --- | --- | --- | --- | --- | --- |
|  | | | | | | |
| 1. **Advance Care Planning About Future Care at the End of Life** | | | | | | |
|  | | | | | | |
|  | | | | | | |
| 1. Advance care planning on end of life care should be initiated at the time of diagnosis of dementia | 1 | 2 | 3 | 4 | 5 | 0 |
|  | | | | | | |
| 1. The process of advance care planning should involve revisiting plans with the patient and the family on a highly frequent basis | 1 | 2 | 3 | 4 | 5 | 0 |
|  | | | | | | |
| 1. When a patient cannot participate in treatment decisions an advance directive is essential | 1 | 2 | 3 | 4 | 5 | 0 |
|  | | | | | | |
| 1. The pace of advance care planning is primarily determined by patient’s and family’s willingness to face the end of life | 1 | 2 | 3 | 4 | 5 | 0 |
|  | | | | | | |
| 1. Families and patients who are involved in advance care planning should become informed about commonly occurring health problems associated with severe dementia, such as pneumonia and intake problems | 1 | 2 | 3 | 4 | 5 | 0 |
|  | | | | | | |
| 1. In the case of severe dementia, the patient’s best interest may be increasingly served with a primary goal of maximising comfort | 1 | 2 | 3 | 4 | 5 | 0 |
|  | | | | | | |
| 1. The physician should take the initiative to introduce and encourage advance care planning | 1 | 2 | 3 | 4 | 5 | 0 |
|  | | | | | | |
| 1. There should be an agreed format for advance care plans | 1 | 2 | 3 | 4 | 5 | 0 |
|  | | | | | | |
| 1. Physicians need improved knowledge to successfully involve families in caring for dementia patients at the end of life | 1 | 2 | 3 | 4 | 5 | 0 |
|  | | | | | | |
| 1. The advance care planning process requires my making family members agree with the physician on goals of care | 1 | 2 | 3 | 4 | 5 | 0 |
|  | | | | | | |
| 1. The physician cannot make family members accept their loved one’s prognosis, the advance care planning process fails | 1 | 2 | 3 | 4 | 5 | 0 |
|  | | | | | | |
| 1. When family members have difficulty understanding the limitations and complications of life sustaining therapies, the physician cannot successfully guide the advance care planning process | 1 | 2 | 3 | 4 | 5 | 0 |

|  | **Strongly disagree** | **Moderately disagree** | **Neither agree nor disagree** | **Moderately agree** | **Strongly agree** | **Don’t know** |
| --- | --- | --- | --- | --- | --- | --- |
|  | | | | | | |
| 1. **Decision-Making** | | | | | | |
|  | | | | | | |
|  | | | | | | |
| 1. Shared decision making including the patient and family caregiver as partners should be a clinical practice goal | 1 | 2 | 3 | 4 | 5 | 0 |
|  | | | | | | |
| 1. The health care provider should always prioritize the patient’s needs in decision making | 1 | 2 | 3 | 4 | 5 | 0 |
|  | | | | | | |
| 1. The physician should be responsible for making the final decision on the patient’s needs | 1 | 2 | 3 | 4 | 5 | 0 |

**Section B: Aspects of Palliative Care in Dementia**

Please consider the following aspects of palliative care in dementia and place a score from 0 to 10 in each cell of the grid to indicate your opinion of their importance, the barriers to achieving optimal care and the amount of effort needed to address each barrier.

| **Aspect of palliative care in dementia** | **How important is this to palliative care in dementia?**  0 = Not important  10 = Very important | **How significant is this as a barrier in your clinical practice?**  0 = Not significant  10 = Very significant | **To what extent does addressing this barrier require effort in terms of time and cost, for you, the institution or national level**  0 = No effort  10 = A lot of effort |
| --- | --- | --- | --- |
|  | | |  |
| 1. Acceptance amongst **professionals** that palliative care applies to dementia |  |  |  |
|  | | |  |
| 1. Acceptance amongst **the public** that palliative care applies to dementia |  |  |  |
|  | | |  |
| 1. Person-centred palliative care in dementia involving optimal communication and shared decision making |  |  |  |
|  | | |  |
| 1. Setting care goals as part of producing advance care plans |  |  |  |
|  | | |  |
| 1. Continuity within palliative care in dementia |  |  |  |
|  | | |  |
| 1. Accurate prognosis to allow for timely recognition of dying |  |  |  |
|  | | |  |
| 1. Minimising aggressive, burdensome, or futile treatment that will not extend life or provide comfort. |  |  |  |
| 1. Treatment and care of symptoms that is designed to provide comfort |  |  |  |
| 1. Psychological and spiritual support |  |  |  |
| 1. Family involvement and associated support for families in caring for the patient |  |  |  |
| 1. Education and training specific to palliative care in dementia for the health care team |  |  |  |
| 1. Availability to specialist support in palliative care for dementia |  |  |  |

**Section C: Barriers and Solutions**

Finally, in your opinion, what are the **three most significant barriers** to providing good quality palliative care in dementia in your practise, and importantly, how would you suggest these barriers are best addressed?

| **Barrier** | **How best might this be addressed?** |
| --- | --- |
| ***Example:*** the inconsistent use of the term palliative care among and between physicians and carers | ***Example:*** multidisciplinary training on site |
| **1.** |  |
| **2.** |  |
| **3.** |  |

**BLANK PAGE**

**Section D Some Questions About You Will Help Our Analyses**

1. Please indicate today’s date: ________________
2. Please indicate your gender:

□ Male

□ Female

1. Please indicate your age: ________________ years
2. How long have you practiced as a physician? ________________ years
3. What proportion of your time is spent providing clinical care?

Please indicate a proportion between 0 FTE to 1.0 FTE (full-time equivalent) ________________

1. What percentage of your practice time involves clinical care in the nursing home?

□ None

□ < 10%

□ 10 - 24%

□ 25 - 49%

□ 50 - 74%

□ 75 - 90%

□ > 90%

1. How often do you visit a typical nursing home patient?

□ at least Daily

□ at least Weekly

□ at least Monthly

□ Every 2 months

□ Every 6 months

□ Less than every 6 months

□ Never

1. Please estimate the number of dying dementia patients you cared for in the past year.

□ None

□ 1 to 4

□ 5 to 9

□ 10 to 19

□ 20 or more

**Thank You for Participating in This Survey**

**Please return the completed survey using the addressed and postage paid envelope**

**If you have any comments or questions please contact**

**Professor Kevin Brazil, Professor of Palliative Care, School of Nursing and Midwifery**

**Queens’s University Belfast, Medical Biology Centre, 97 Lisburn Road, Belfast BT9 7BL**

**Tel: 028 9097 5782 Email:** [**k.brazil@qub.ac.uk**](mailto:k.brazil@qub.ac.uk)
